# Development and evaluation of a jaw-tracking system for mice: reconstruction of three-dimensional movement trajectories on an arbitrary point on the mandible

**DOI:** 10.1186/s12938-019-0672-z

**Published:** 2019-05-16

**Authors:** Emi Moriuchi, Ryo Hamanaka, Yoshiyuki Koga, Ayumi Fujishita, Tomoko Yoshimi, Go Yasuda, Haruka Kohara, Noriaki Yoshida

**Affiliations:** 1 Department of Orthodontics and Dentofacial Orthopedics, Graduate School of Biomedical Sciences, Nagasaki University, 1-7-1 Sakamoto, Nagasaki, 852-8588 Japan; 2 Department of Orthodontics, Nagasaki University Hospital, 1-7-1 Sakamoto, Nagasaki, 852-8588 Japan

**Keywords:** Motion capture, Jaw movements, Mastication, Mouse, Rigid transformation

## Abstract

**Background:**

Mastication is one of the most fundamental functions for the conservation of life. The demand for devices for evaluating stomatognathic function, for instance, recording mandibular movements or masticatory muscle activities using animal models, has been increasing in recent years to elucidate neuromuscular control mechanisms of mastication and to investigate the etiology of oral motor disorders. To identify the fundamental characteristics of the jaw movements of mice, we developed a new device that reconstructs the three-dimensional (3D) movement trajectories on an arbitrary point on the mandible during mastication.

**Methods:**

First, jaw movements with six degrees of freedom were measured using a motion capture system comprising two high-speed cameras and four reflective markers. Second, a 3D model of the mandible including the markers was created from micro-computed tomography images. Then, the jaw movement trajectory on the certain anatomical point was reproduced by integrating the kinematic data of the jaw movements with the geometric data of the mandible.

**Results:**

The 3D movements at any points on the mandible, such as the condyle, molar, and incisor during mastication, could be calculated and visualized with an accuracy > 0.041 mm in 3D space. The masticatory cycle was found to be clearly divided into three phases, namely, the opening, closing, and occlusal phases in mice.

**Conclusions:**

The proposed system can reproduce and visualize the movements of internal anatomical points such as condylar points precisely by combining kinematic data with geometric data. The findings obtained from this system could facilitate our understanding of the pathogenesis of eating disorders or other oral motor disorders when we could compare the parameters of stomatognathic function of normal mice and those of genetically modified mice with oral behavioral dysfunctions.

## Background

Mastication is one of the most common rhythmic behaviors along with respiration and locomotion, and a fundamental function for the maintenance of life. To elucidate the central and peripheral control mechanisms of masticatory function, oral motor behaviors have been investigated using animals such as rabbits [1–3], guinea pigs [4, 5], cats [6, 7], mice [8], and rats [9, 10].

Recent developments in molecular biology have led to the creation of genetically modified mice expressing various kinds of oral dysfunctions, such as serotonin receptor-deficient mice [11] that present with eating disorders and epilepsy. Comparisons between normal mice and genetically modified mice with oral motor disorders will enhance our understanding of neural and motor disorders in the craniomandibular system. Given this background, the demand for mice to use as animal models for identifying the etiology of oral motor disorders is increasing.

Although several attempts have been made to record jaw movements of small animals by means of optoelectronic measurement systems [5, 9] and electromagnetic measurement systems [8, 12, 13], majority of them were limited to the measurement of movements at only one external point on the mandible. In addition, there have been very few studies to evaluate oral functions in mice due to the difficulties in mounting the instrument on such small animals and in measuring a very small range of jaw movement within 2 mm cube.

Movements of one target point could never provide the whole aspect of jaw movement, since any anatomical points on the mandible are going to move in different directions like a rigid body motion with six degrees of freedom. To elucidate the fundamental characteristics of the jaw movements and the role of muscles associated with masticatory function in mice, movements of arbitrary points on the mandible, namely, anatomical landmark points, such as molar and condylar points, have to be detected.

The purpose of this study was to describe and evaluate a newly developed approach to measure jaw movements with six degrees of freedom and to visualize and quantify the movements of internal anatomical points such as condylar points by integrating kinematic data obtained from a motion capture system [14–16] with geometric data of the mandible created from micro-computed tomography (micro-CT) images [17] during mastication in mice.

## Methods

### System description

To reproduce dynamic jaw movement trajectories and analyze the movements of arbitrary points on the mandible during mastication, two kinds of data were acquired. One was the kinematic data obtained from a motion capture system, and the other was the geometric data of the mandible created from images scanned with an in vivo micro-CT system (R-mCT, Rigaku, Japan). The kinematic data of the jaw movements were combined with the geometric data of the mandible to detect the 3D movements at any points on the mandible, such as the condylar point, the incisal edge on the anterior tooth, and the cusp of the molar.

### Acquisition of kinematic data

**Fig. 1:**
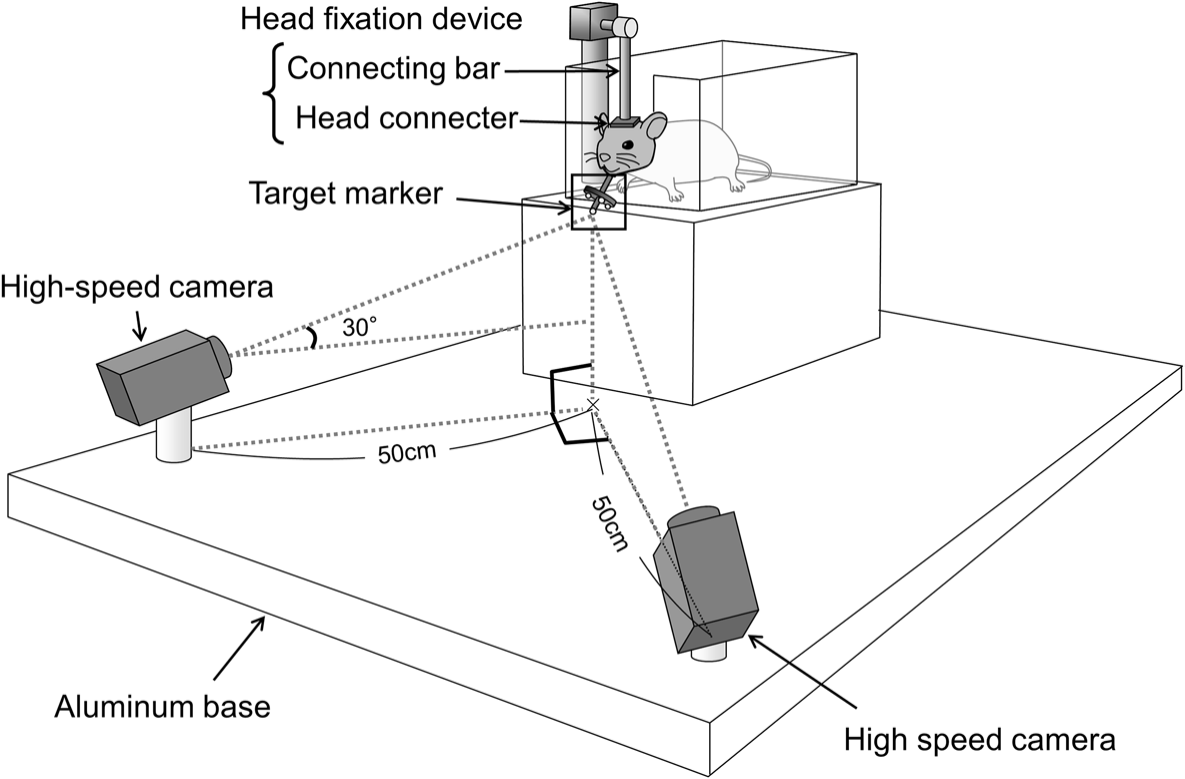
Schematic illustration of the motion capture system. Two high-speed cameras were orthogonally positioned 50 cm away from the target markers and directed obliquely upward by 30°. Four reflective markers were attached to the mandible of the mouse whose movement was restrained using the head fixation device

Jaw movements during mastication were recorded using a motion capture system (DIPP-Motion V3D, DITECT, Japan). The jaw-tracking system consisted of two high-speed cameras (HAS220, DITECT), four reflective target markers (Alumina ball HD, Nikkato, Japan), a head fixation device including a head connector (female IC socket) and a connecting bar, and an aluminum base on which the cameras are fixed (Fig. 1). The two high-speed cameras were used to estimate the position of the four target markers based on triangulation. The cameras were orthogonally positioned 50 cm away from the target markers. Each camera was directed obliquely upward toward the target markers to capture all four markers during the entire mastication process.

**Fig. 2:**
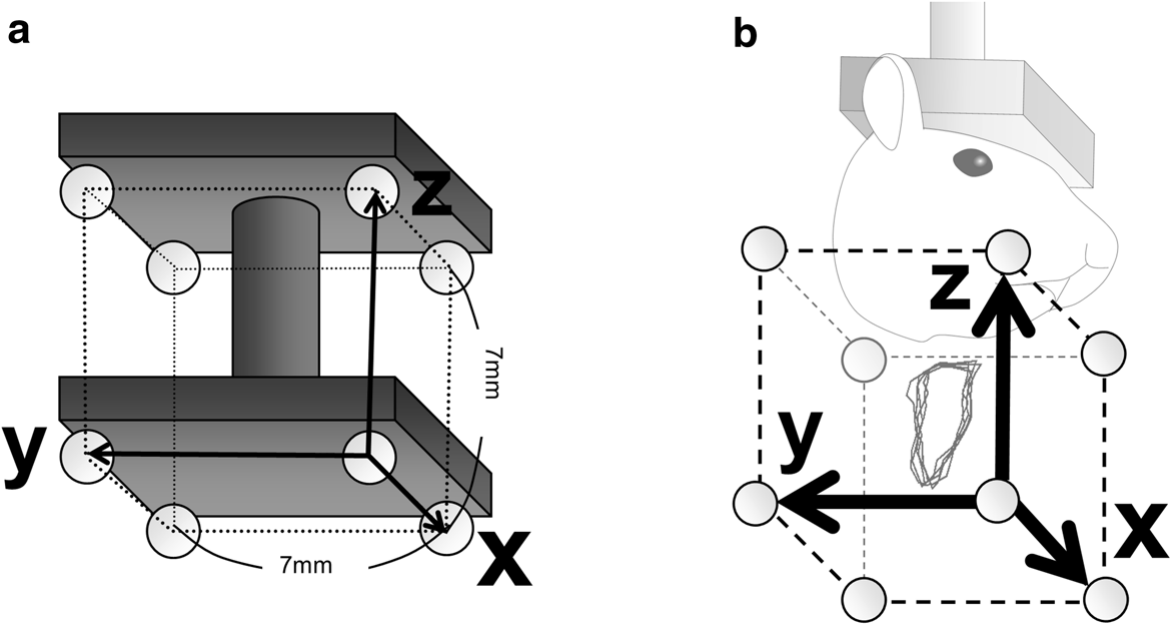
Camera calibration. **a** Calibrator, onto which eight alumina balls as markers arranged at each corner of a 7 mm cube, were attached. **b** Calibrator was placed in an area, where the mouse mandible moves during mastication

Before recording jaw movements, the coordinate system of the motion capture system was established according to the following calibration process. Eight alumina balls fixed to each corner of a 7 mm cube were used to calibrate the camera (Fig. 2a). This calibrator was placed so as to cover the entire range of motion of the markers, while mice are chewing. The direction of each side of the calibrator cube demonstrates the coordinate axis; the *x*-axis represents the transverse direction showing right and left, and the *y*-axis indicates the anterior–posterior direction, which is parallel to the occlusal plane of mice, and the *z*-axis represents the vertical direction (Fig. 2b). Each camera outputted an image of the calibrator. Then, based on triangulation, the two-dimensional coordinates of the eight balls on the two images were transformed into 3D coordinates using DIPP-Motion V3D (DITECT), and the coordinate system of the motion capture system was established (Fig. 2b).

**Fig. 3:**
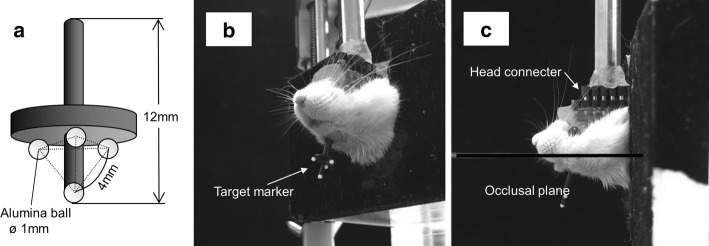
Target marker assembly. **a** Marker assembly, onto which four alumina balls as markers were arranged at each corner of a 4 mm regular triangular pyramid, was attached. **b** Mouse’s head was connected to the fixation device, and the target marker assembly was fixed to the mandible. **c** Occlusal plane of the mouse was set to be parallel to the head connecter and the ground

Jaw movements were recorded by tracking the positions of the target markers. The target marker assembly for the motion capture comprised a frame made of acrylic resin and four target makers of 1-mm-diameter reflective alumina balls, which were attached to each corner of a regular tetrahedron with 4 mm sides (Fig. 3a).

Fifteen-week-old male Jcl:ICR mice (Clea, Japan) were used to confirm the accuracy of our system. To attach the target markers and a connector (male IC socket) for head fixation, the mice were anesthetized by intra-peritoneal injection of 5:1.5:3.5 ketamine (Ketalar, Sankyo Yell, Japan), xylazine (Selactar 2%, Bayer Health-care, Japan), and 0.9% sodium chloride solution. The target markers and the connector were bonded, respectively, to the parietal bones and the lower surface of the mandible using 4-META resin (Super-Bond, Sun Medical, Japan) (Fig. 3b). The head connecter was set parallel to the occlusal plane (Fig. 3c).

Jaw movements were recorded, while the mouse chewed a ball-shaped hard pellet (diameter, 1.5 mm) 3 days after the surgical preparation. The frame rates of the cameras were set to be 200 frames/s, which enables to capture the fast jaw movements of mice with a frequency of approximately 5 Hz. The captured images were binarized to clearly distinguish the markers from the background, and the center points of all markers were tracked.

The experimental protocol of this study was approved by the Animal Welfare Committee of Nagasaki University based on the Animal Care Standards of this institution (approval no. 1401141113-3). Every possible effort was taken to minimize animal suffering.

### Three-dimensional reconstruction of geometric model

After measurement of the jaw movements, micro-CT images of the head were taken to create 3D geometric models, while mice were anesthetized. Slice thickness, tube voltage, and tube current were 0.8 mm, 80 kV, and 100 mA, respectively. The CT images were taken, so that the four target markers and whole mandible were included. Then, the 3D surface of the mandible with the four markers was reconstructed from the CT images using 3D image processing and editing software (Mimics 20.0, Materialise, Belgium). Thus, relative position of the mandible with respect to the four makers was recorded.

### Associating kinematic data with geometric model

**Fig. 4:**
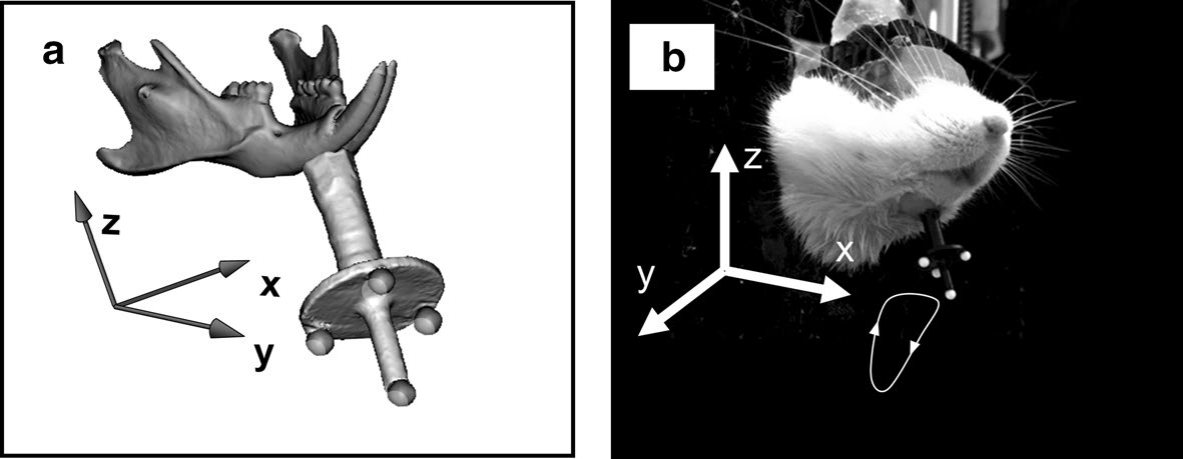
**a** Coordinate system of the geometric model. **b** Coordinate system of the motion capture system

To perform 3D reconstruction of the jaw movements, the kinematic data and the geometric model were registered through the four markers. That is, the coordinate system of the geometric model (Fig. 4a) was converted into the coordinate system of the motion capture system (Fig. 4b) using rigid transformation. The optimal values of a rotation matrix and a translation vector between the coordinates of the four markers in the coordinate system of the geometric model and the corresponding coordinates in the coordinate system of the motion capture system for rigid transformation were estimated.

**Fig. 5:**
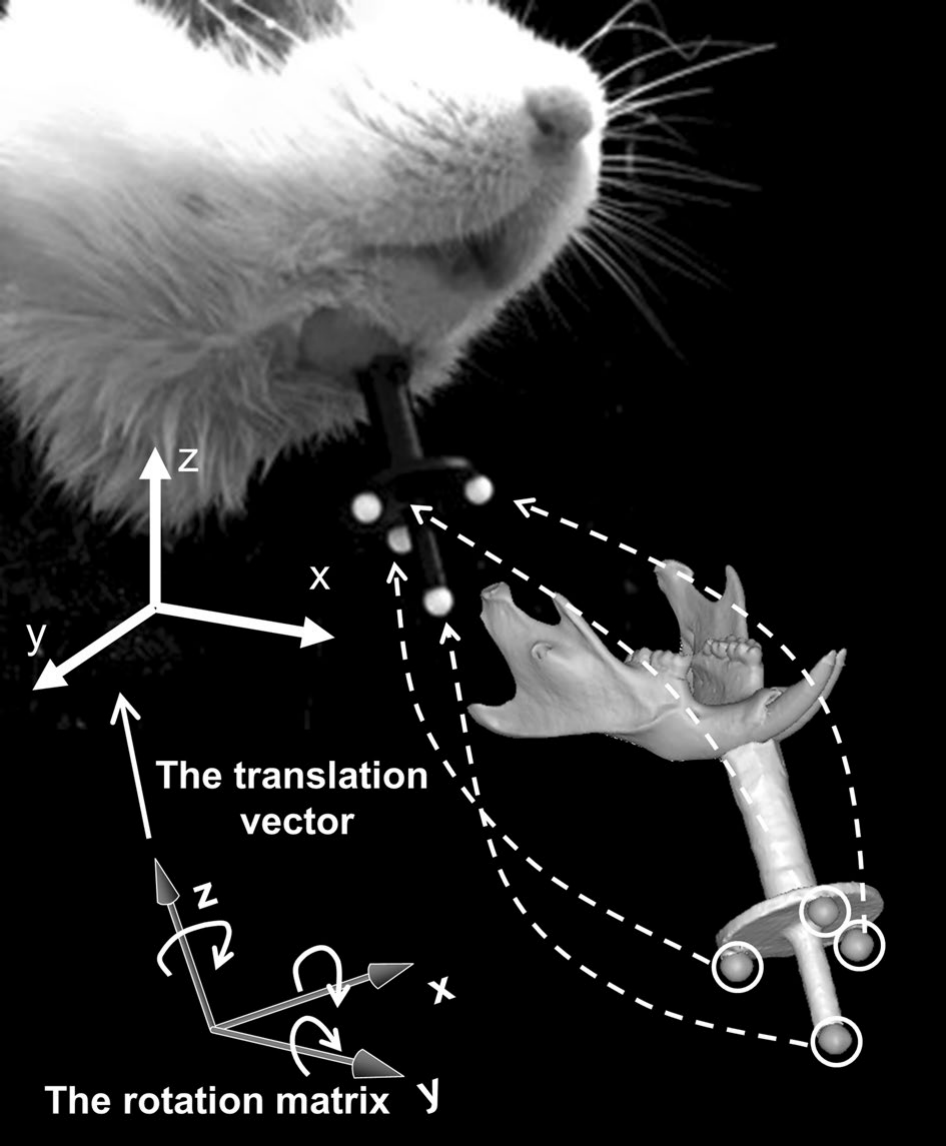
Schematic illustration of rigid transformation. Each of the coordinates of the four markers in the coordinate system of the geometric model is transferred to that in the coordinate system of the motion capture system by means of a rigid transformation

Figure 5 shows a diagram of the rigid transformation in which each of the coordinates of the four markers in the coordinate system of the geometric model is transferred to that in the coordinate system of the motion capture system. This rigid transformation minimizes the sum of squared difference between the coordinate values of four markers at each frame of motion capture data and their corresponding values obtained from the rigid transformation of the geometric model, and it can be determined by minimizing the following least squared error function:

$$ \sum\limits_{i = 1}^{4} {\left\| {R^{t} p_{i} + U^{t} - P_{i}^{t} } \right\|^{2} } , $$

where $P_{i}^{t}$ is a position vector of the $i$th marker ($i\, = \,1,\,2,\,3,\,4$) in the coordinate system of the motion capture system at time $t$, and $p_{i}$ is a position vector of the $i$th marker in the coordinate system of the geometric model of the mandible, which was obtained from reconstructed micro-CT images. $R^{t}$ and $U^{t}$ are a rotation matrix and a translation vector, respectively, which represent the rigid transformation. We determined $R^{t}$ using Horn’s unit quaternion method [18, 19] and computed $U^{t}$ from displacement of the centroid of the four markers.

Then, the coordinate value of an arbitrary point on the geometric model with respect to the coordinate system of the motion capture system at time $t$ can be calculated as follows:

$$ V = R^{t} v + U^{t} , $$

where $v$ is a position vector in the coordinate system of the geometric model reconstructed from the micro-CT image, and $V$ is the corresponding position vector in that of the motion capture system. The 3D movement of an arbitrary point on the mandible can be calculated by inputting the determined rotation matrix and translation vector into the corresponding values in the coordinate system of the motion capture system on the assumption that the relative position between the four markers and the mandible is unchanged and the mandible is a rigid body. In the present study, movements of the right and left condylar points, the mandibular molar cusp points, and an incisal point on the lower incisor were calculated.

### System evaluation

To evaluate the accuracy of measurements of the system, a set of four markers were moved within a 3 mm^3^ region in 0.5 mm steps in 3D space on a three-axis motorized pulse stage (MM-60XY, MM-60V, Chuo Precision Industrial Co., Japan). The difference between the displacement mechanically driven by the motorized pulse stage and the measured displacement using the motion capture system was calculated at each calibration point as the measurement error. The mean measurement errors were 0.041 mm in the *x*-axis, 0.042 mm in the *y*-axis, and 0.034 mm in the *z*-axis (Table 1).

**Table 1:** Measurement error (mm) of the system

	Mean	Standard deviation	Maximum error
*x*-axis	0.041	0.029	0.138
*y*-axis	0.042	0.027	0.126
*z*-axis	0.034	0.034	0.108

**Fig. 6:**
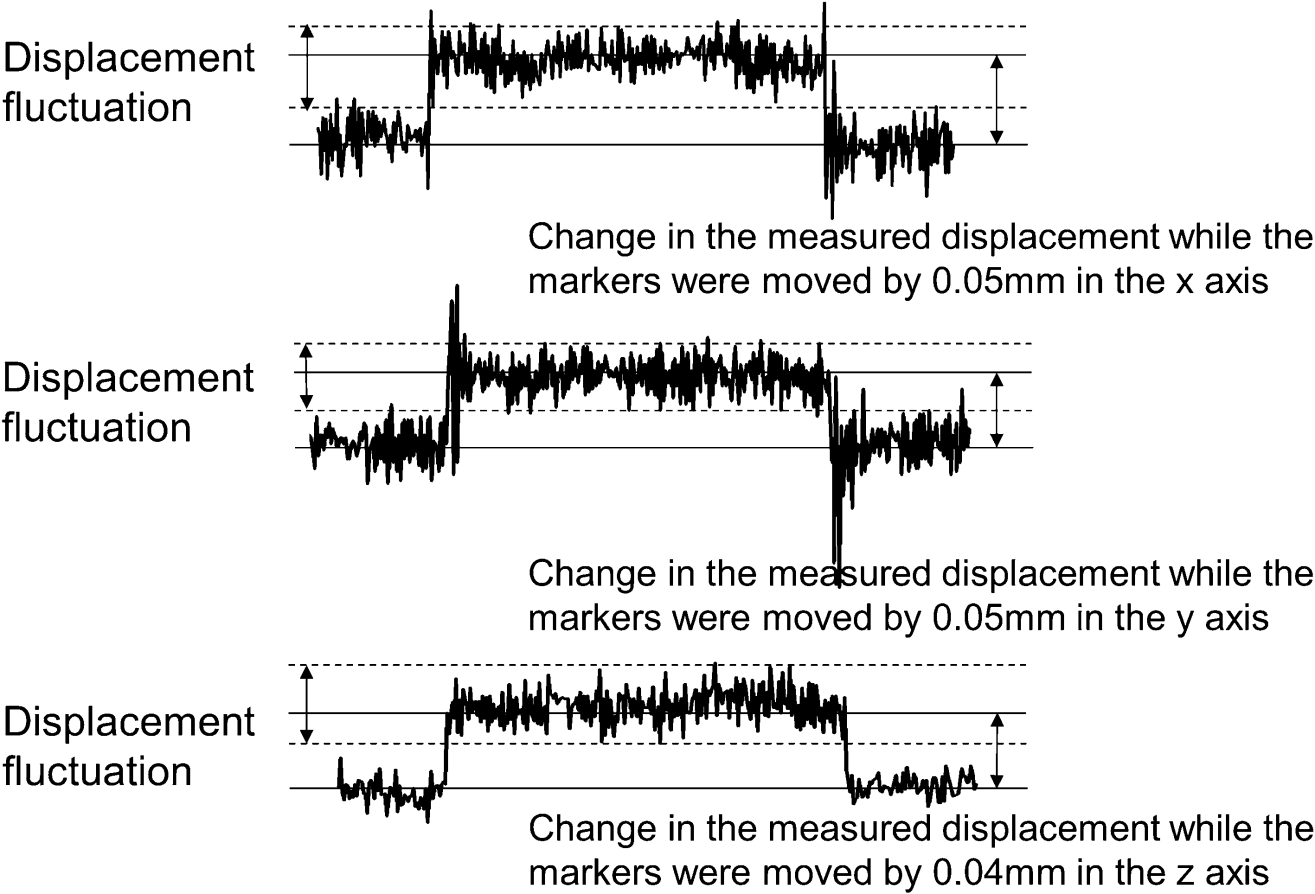
Resolution of the system for displacement. The target marker was moved along the *x*-axis (top), *y*-axis (middle), and *z*-axis (bottom)

To determine the resolution for displacement, displacements were measured, while the target marker on the stage was moved along the *x*- and *y*-axes separately by 0.05 mm, and along the *z*-axis by 0.04 mm. As a result, the resolution was found to be < 0.05 mm for displacement in the *x*- and *y*-axes, and 0.04 mm for the *z*-axis, since the change in the measured displacement was larger than the range of the displacement fluctuation in all cases (Fig. 6).

To evaluate the repeatability of measurement, the intraclass correlation coefficient (ICC) and 95% confidence interval (CI) were used [20, 21]. The values indicating the vertical excursion of movement trajectories of the incisor during ten successive chewing were collected from three mice for two times. Then, ICC and 95% CI were calculated, as shown in Table 2.

**Table 2:** Mean ± standard deviation of vertical excursion (mm), repeatability (ICC, %), and 95% CI (*n* = 3)

	Repetition 1	Repetition 2
Mouse 1	3.861 ± 0.126	3.804 ± 0.282
Mouse 2	3.861 ± 0.361	4.164 ± 0.146
Mouse 3	4.182 ± 0.173	4.282 ± 0.203

ICC	95% CI	
	Lower	Upper
0.641	− 0.557	0.989

## Results

**Fig. 7:**
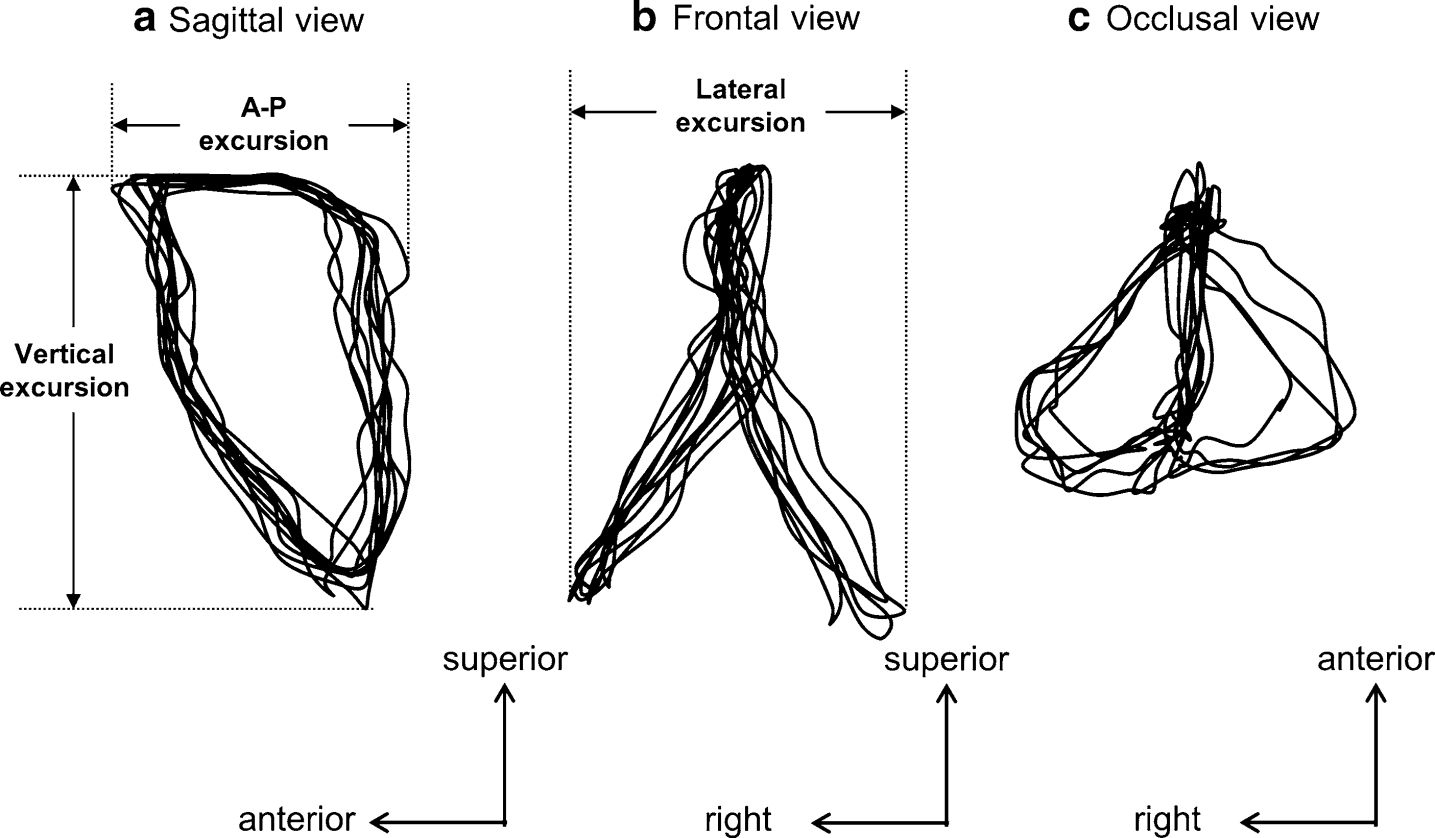
Movement trajectories of the molar projected on the **a** sagittal, **b** frontal, and **c** occlusal planes. Jaw movement parameters measures ware vertical excursion, A–P excursion, and lateral excursion

**Fig. 8:**
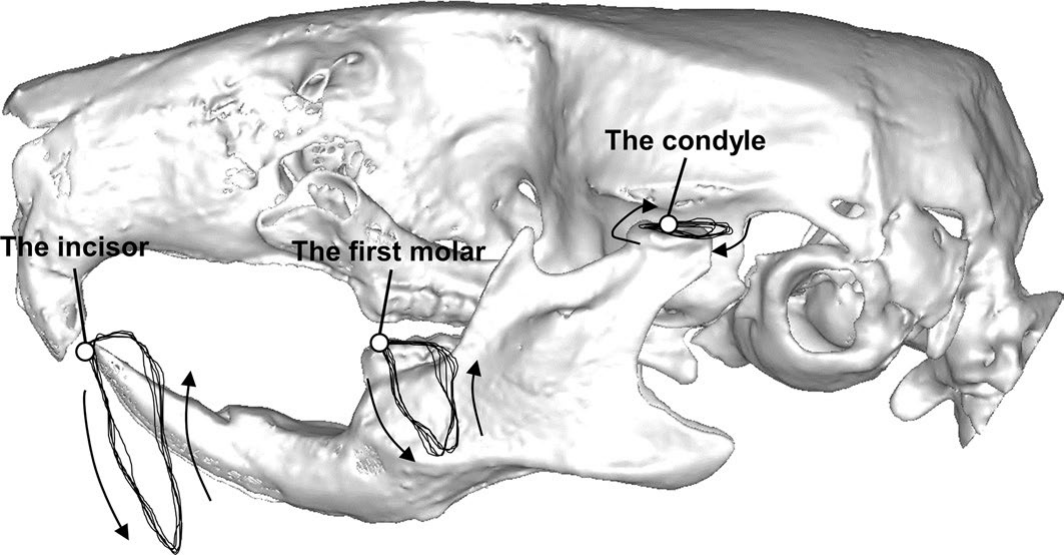
Paths of three-dimensional jaw movements on analyzed points of the incisal edge, and the molar cusps and the condyles on the working and balancing sides were reconstructed and projected on the sagittal plane

**Fig. 9:**
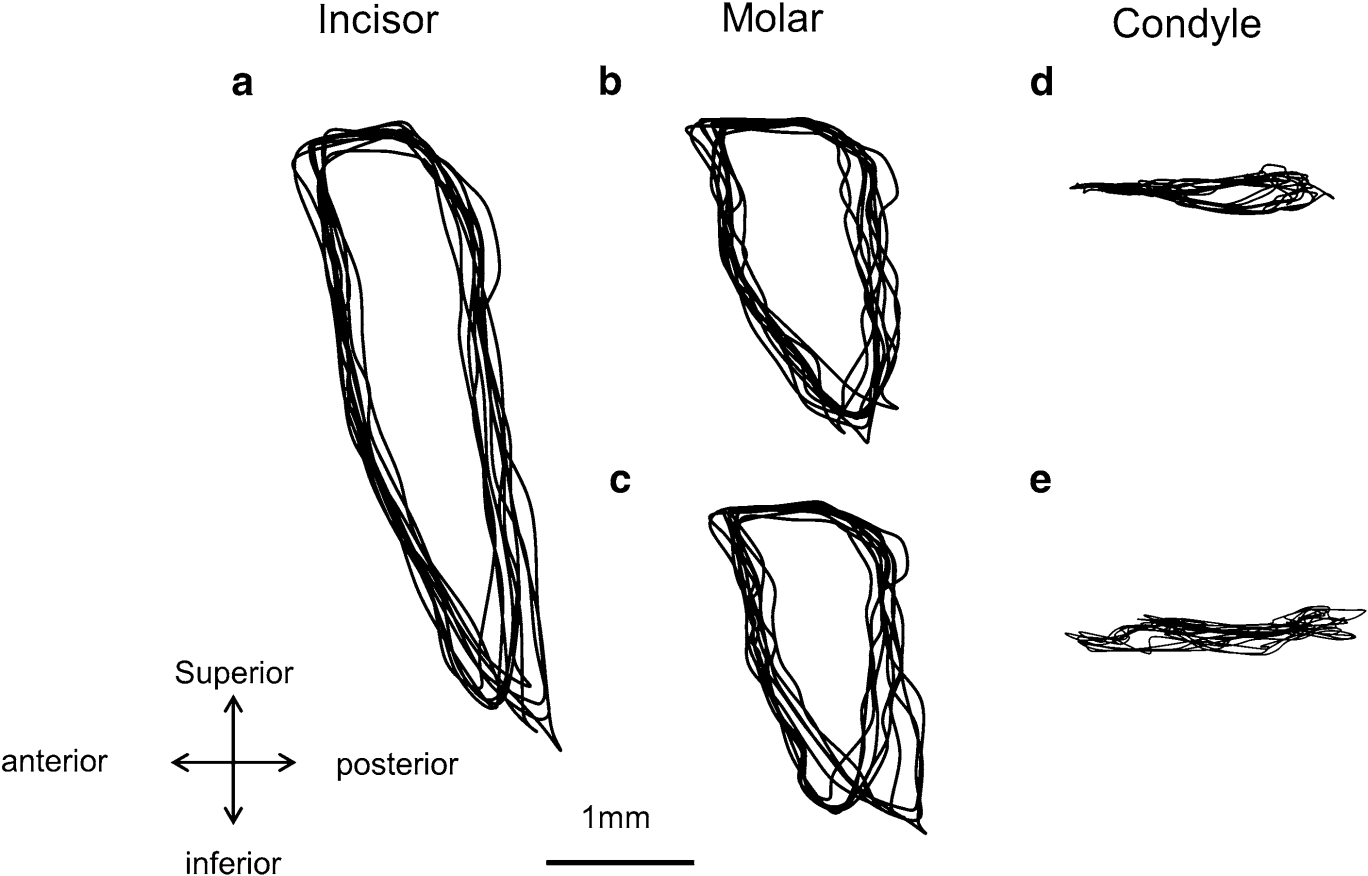
Lateral views of five successive chewing cycles linked to each of five analyzed points. **a** Incisor, **b** molar on the working side, **c** molar on the balancing side, **d** condyle on the working side, and **e** condyle on the balancing side

**Fig. 10:**
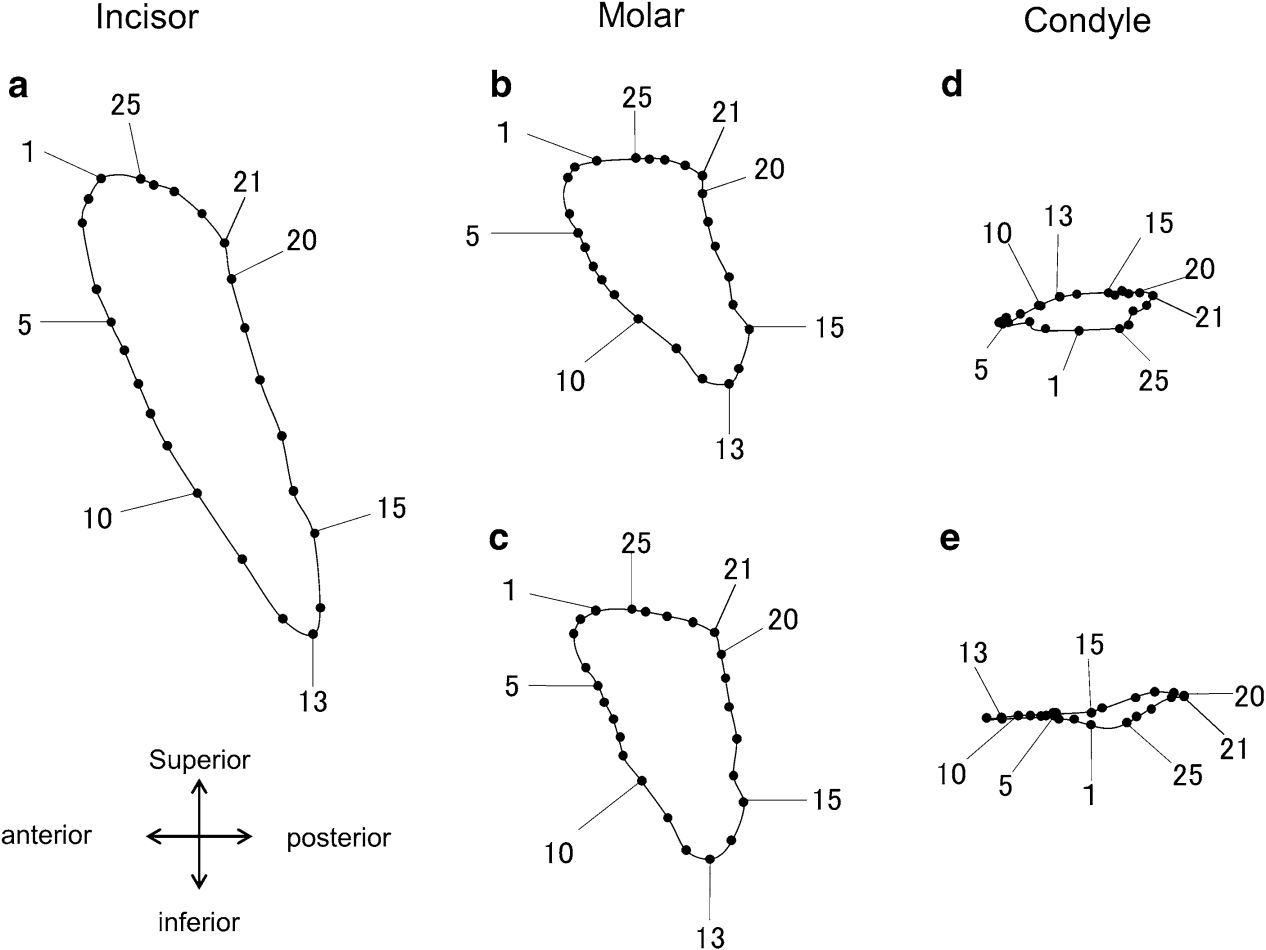
Jaw movement trajectories of one chewing cycle on which coordinates of analyzed points for each frame of motion capture data were plotted. **a** Incisor, **b** molar on the working side, **c** molar on the balancing side, **d** condyle on the working side, and **e** condyle on the balancing side

The paths of 3D jaw movements of the incisor, molar, and condyle were reconstructed. To cite an example, the movement trajectories of the molar were reconstructed and projected on the sagittal, frontal, and occlusal planes, respectively, as shown in Fig. 7. Figure 8 shows the lateral views of ten successive chewing cycles linked to each analyzed point of the incisor, molar, and condyle. Figure 9 shows the movement trajectories of the incisor, and the molar cusps and the condyles on the working and balancing sides projected on the sagittal plane. To visualize the velocity of the jaw movements at each timepoint and to set a point on a trajectory of one analyzed point in correspondence to another, coordinates of analyzed points for each frame of motion capture data were plotted on the jaw movement trajectories of one masticatory cycle (Fig. 10). Since the video images were taken at intervals of 5 ms, the distance from one point to the next indicates the velocity of the movement.

Table 3 shows the mean values and standard deviation (SD) of vertical excursion, antero-posterior (A–P) excursion and lateral excursion of movement trajectories of the incisor and molars during ten successive chewing.

**Table 3:** Jaw movement parameters during ten successive chewing (mm)

	Incisor	Molar (right)	Molar (left)
Vertical excursion	3.861 ± 0.126	1.972 ± 0.072	1.966 ± 0.058
A–P excursion	1.413 ± 0.130	1.175 ± 0.048	1.166 ± 0.131
Lateral excursion	2.103 ± 0.196	1.331 ± 0.155	1.300 ± 0.152

The mean values and standard deviation (SD) of vertical excursion, antero–posterior (A–P) excursion and lateral excursion of movement trajectories of the incisor and molars during ten successive chewing

Table 4 shows the A–P excursion of movement trajectories of the condyle on the working side and balancing side.

**Table 4:** A–P excursion of the movement trajectories of the condyle (mm)

	A–P excursion
WS	1.027 ± 0.066
BS	1.479 ± 0.066

A–P excursion of the movement trajectories of the condyle on the working side (WS) and the balancing side (BS)

Regarding the movement trajectory of the incisor, the opening movements showed a backward depression in the sagittal plane. The closing movement consisted of anterior and superior movements. The mandible traced a more posterior path in the closing phase than in the opening phase in mice unlike in humans.

Regarding the movement trajectory of the molar, the masticatory cycle was found to be clearly divided into three phases, the opening (from timepoints 1 to 13), closing (from timepoints 13 to 21), and occlusal phases (from timepoints 21 to 1), as shown in Fig. 10. Based on the movement trajectory of the molar, we determined that the mandibular position at timepoint 1 was the maximum jaw-closing position, that at timepoint 13 was the maximum jaw-opening position, and that at timepoint 21 was the beginning of the occlusal phase. The occlusal phase was characterized by the forward movement, and the molar moved more parallel to the occlusal plane on the working side than on the balancing side.

Regarding the movement trajectory of the condyle, the masticatory cycle showed anterior and posterior movements that were nearly straight and parallel to the occlusal plane. The condyle on the working side began the forward movement at its most posterior position (Fig. 10d, timepoint 21), and further moved anteriorly until the maximum jaw-closing position (timepoint 1) during the occlusal phase. It further moved anteriorly, and then, interestingly, its direction of movement changed from anterior to posterior midways through the jaw-opening phase. On the other hand, the condyle on the balancing side moved anteriorly throughout the opening phase. Table 4 also shows a larger A–P excursion in the balancing side than the working side.

## Discussion

We developed a novel device that can measure jaw movements with six degrees of freedom in mice. The 3D movements at any points on the mandible, for instance, points of the condyle, molar and incisor could be calculated through integration and registration of the kinematic data of the jaw movements and the geometric data of the mandible on four markers.

In the previous studies [1, 9, 22], measuring devices could detect movements of only one target point that was attached to the mandible as an external landmark. However, the advantage of the system was to have the capability to measure jaw movements in freely moving mice. On the other hand, the jaw movement had to be somewhat restrained under the non-physiological condition when using the method developed in the present study. Nevertheless, this method has a great advantage to have the capability to reproduce and visualize the movements of internal anatomical points such as condylar points precisely by superimposing the micro-CT images of the mandible on each frame of images captured by two high-speed cameras during mastication.

As a result, the characteristics of the jaw movements of mice during mastication could be evaluated in greater detail. Fast jaw movements of mice with a frequency of approximately 5 Hz could be efficiently recorded using the high-speed cameras that can capture images at 200 frames per second. The measured chewing trajectories clearly demonstrated the characteristics of the jaw movements of mice. Images of all markers on the mandible were captured throughout the recording of dynamic jaw movements by positioning the two cameras orthogonally.

For the purpose of acquiring micro-CT images of the mandible with high resolution, acrylic resin (non-metallic material) was used for the base material, to which the target 1-mm-diameter ball markers were attached, to minimize artifacts. On the CT image, the alumina balls as the target markers could be easily discriminated in the base resin due to the difference in the CT value between the resin and the alumina. Furthermore, the marker assembly was light, weighing only 0.1 g, which was less likely to obstruct the physiological jaw movements of the mice during mastication. Since the mean measurement errors were 0.041 mm in the *x*-axis, 0.042 mm in the *y*-axis, and 0.034 mm in the *z*-axis, the accuracy of the system was found to be high enough to measure the jaw movements of mice, whose gape size ranges from 1.5 to 2.0 mm. A displacement resolution was calculated to be less than 0.05 mm for displacement in the *x*- and *y*-axes, and 0.04 mm for the *z*-axis.

The value of ICC indicating the repeatability of the measurement was 0.641. Since ICC was interpreted as poor (< 0.4), fair (0.4–0.6), good (0.6–0.75), and excellent (≥ 0.75) [23], the repeatability was found to be high enough to measure the jaw movements of mice.

The system showed a reasonable performance for practical measurement of the jaw movements of small animals as compared with the previously developed devices [12, 24]. For these reasons, this system was found to be sufficiently stable and reliable to measure the jaw movements of mice.

The movement trajectories of the incisor demonstrated that the jaw-opening and jaw-closing paths diverge widely in the sagittal plane unlike in humans. Anatomic studies indicate that the temporalis has a more posteriorly oriented force vector, whereas that of the masseter is oriented more anteriorly [25]. It is, therefore, suggested that characteristic masticatory jaw movements of mice are mainly formed by the motor coordination of the masseter and temporalis muscles [26]. Based on the movement trajectories of the molar, the masticatory cycle could be clearly divided into three phases: opening, closing, and occlusal phases. This indicates that mice grind the food bolus by sliding the posterior teeth from back to front. The movement trajectories of the condyle revealed a unique characteristic. In the late-opening phase, the bilateral condyles moved asymmetrically, although they moved similarly in the early opening phase. That is, the condyle on the working side moved anteriorly, and the condyle on the balancing side moved posteriorly in the late-opening phase. This asymmetric condyle movement likely caused the lateral shift of the mandible in the opening phase. Thus, movement trajectories on internal anatomical points on the mandible during mastication, which were detected using the newly developed system, would enhance our understanding of the mechanism of mastication and motor coordination of masticatory muscles on the right and left sides in mice.

Further attempts to record masticatory muscle electromyograms synchronously with measurements of the jaw movements of mice are needed to elucidate the mechanism of motor coordination of these muscles and their roles during mastication. Fully investigating the stomatognathic function in genetically modified mice with different kinds of oral dysfunctions will greatly enhance our understanding of neural and motor disorders in the craniomandibular system.

## Conclusion

This study provides a description of a method to measure jaw movements with six degrees of freedom and to analyze 3D movements of arbitrary points on the mandible during mastication in mice. The advantage of the system is that it can reproduce and visualize the movements of internal anatomical points such as condylar points precisely by integrating kinematic data obtained from a motion capture system with geometric data of the mandible created from micro-CT images. Obtained data could facilitate our understanding of the molecular genetic pathology of oral motor disorders by making comparisons between normal mice and genetically modified mice with behavioral dysfunctions in the maxillofacial region.
